# Living with Dementia in Kenya: Pathways to Care, Diagnosis and Management

**DOI:** 10.1002/alz70858_098462

**Published:** 2025-12-25

**Authors:** Edna N Bosire

**Affiliations:** ^1^ Aga Khan University, Nairobi, Kenya

## Abstract

**Background:**

Sub‐Saharan Africa (SSA) is experiencing a sharp rise in the size of the older population; consequently, the incidence and prevalence of Alzheimer's Disease Related Dementias (AD/ADRD) is also expected to rise. There is limited research on AD/ADRD lived experiences have been conducted in SSA including Kenya. Our project aimed to explore patient's and caregivers lived experiences with Alzheimer's disease in Kenya.

**Methods:**

This ethnographic study was conducted at the Aga Khan University hospital (AKUH), involving in‐depth interviews with 21 health care providers, 26 patients with mild cognitive impairment and 36 caregivers. We also conducted 10 home visits and observations. Data were transcribed verbatim and thematically analysed. In what follows, we focus on vignettes of two people living with AD in Kenya, and their caregivers. Data from the other interviews are woven into the two vignettes to demonstrate lived experiences of AD.

**Results:**

We identified 5 key themes: Knowledge and perception of dementia; Care pathways for patients; Dementia screening and diagnosis; Social support systems; Challenges and opportunities living with dementia. Overall, patients with dementia in Kenya have unique experiences – based their social‐cultural and economic status, and access to healthcare services. Children or spouses are primary caregiver, and most reported caregiving burden.

**Conclusions & recommendations:**

People's lived experiences with AD in Kenya is unique and influenced by multiple factors. Training primary health care providers in dementia screening and diagnosis, ensuring availability of diagnostic tools, increasing advocacy and awareness of dementia in Kenya are essential steps to enhance timely care seeking, screening, diagnosis and treatment. More research is needed to develop evidence‐based community interventions to support informal care provision for persons with dementia in Kenya.

**Keywords**: Dementia, Lived experiences, Vignettes; Diagnosis, Treatment, Kenya.

